# New risk score for predicting progression of membranous nephropathy

**DOI:** 10.1186/s12967-019-1792-8

**Published:** 2019-02-08

**Authors:** Hu Xiaofan, Xu Jing, Gao Chenni, Wu Yifan, Yu Xialian, Lin Li, Ren Hong, Zhang Wen, Wang Weiming, Pan Xiaoxia, Xie Jingyuan, Chen Nan

**Affiliations:** 1 Department of Nephrology, Institute of Nephrology, Shanghai Ruijin Hospital, Shanghai Jiao Tong University, School of Medicine, Shanghai, 200001 China; 2 Insititution of KECC, University of Michigan, Ann Arbor, USA

**Keywords:** Chronic kidney disease, Membranous nephropathy, Risk score, Prognosis

## Abstract

**Background:**

Patients with Idiopathic membranous nephropathy (IMN) have various outcomes. The aim of this study is to construct a tool for clinicians to precisely predict outcome of IMN.

**Methods:**

IMN patients diagnosed by renal biopsy from Shanghai Ruijin Hospital from 2009.01 to 2013.12 were enrolled in this study. Primary outcome was defined as a combination of renal function progression [defined as a reduction of estimated glomerular filtration rate (eGFR) equal to or over 30% comparing to baseline], ESRD or death. Risk models were established by Cox proportional hazard regression analysis and validated by bootstrap resampling analysis. ROC curve was applied to test the performance of risk score.

**Results:**

Totally 439 patients were recruited in this study. The median follow-up time was 38.73 ± 19.35 months. The enrolled patients were 56 (15–83) years old with a male predominance (sex ratio: male vs female, 1:0.91). The median baseline serum albumin, eGFR-EPI and proteinuria were 23(8–43) g/l, 100.31(12.81–155.98) ml/min/1.73 m^2^ and 3.98(1.50–22.98) g/24 h, respectively. In total, there were 36 primary outcomes occurred. By Cox regression analysis, the best risk model included age [HR: 1.04(1.003–1.08), 95% CI from bootstrapping: 1.01–1.08), eGFR [HR: 0.97 (0.96–0.99), 95% CI from bootstrapping: 0.96–0.99) and proteinuria [HR: 1.09 (1.01–1.18), 95% CI from bootstrapping: 1.02–1.16). One unit increasing of the risk score based on the best model was associated with 2.57 (1.97–3.36) fold increased risk of combined outcome. The discrimination of this risk score was excellent in predicting combined outcome [C statistics: 0.83, 95% CI 0.76–0.90].

**Conclusions:**

Our study indicated that older IMN patients with lower eGFR and heavier proteinuria at the time of renal biopsy were at a higher risk for adverse outcomes. A risk score based on these three variables provides clinicians with an effective tool for risk stratification.

**Electronic supplementary material:**

The online version of this article (10.1186/s12967-019-1792-8) contains supplementary material, which is available to authorized users.

## Background

Idiopathic membranous nephropathy (IMN) is one of the most common types of adult-onset primary glomerulonephritis [1–3]. The incidence of IMN has increased dramatically recently at least in China [2, 4, 5] which maybe partly due to air pollution for example the increased level of PM2.5 in the air [5]. IMN is an immune complex-mediated glomerular disease. The understanding of the pathophysiological mechanism underlying IMN has been greatly improved thanks to the discovery of anti-PLA2R and anti-THSD7A antibodies in IMN patients [6, 7]. Interestingly, previous studies [8–10] based on western population have shown that the level of anti-PLA2R antibody in serum was helpful in the differential diagnosis and the prognosis prediction.

It was reported that approximately one-third of all IMN patients will develop end stage renal disease (ESRD). Both clinical variables including age, gender, serum creatinine, proteinuria and histological variables including tubulointerstitial fibrosis and focal segmental sclerosis(FSGS) at time of diagnosis were associated with renal function progression in IMN patients based on prior studies [11, 12]. However, Trayanov et al. [13] failed to validate the correlation between FSGS and progressive renal disease. Zent et al. [14] found that elderly and young patients had similar rates of ESRD (12% vs 18%, P > 0.05) based on a cohort of 323 IMN patients. The discrepant findings suggested validating studies were necessary in independent cohorts with diverse populations since most of these studies were performed in Western countries. Finally, establishing a risk model to combine the independent predictors could potentially improve the accuracy of prediction since the effect of each single predictor is relatively small. In this study, we enrolled an extended Chinese IMN cohort to establish a risk score to precisely predict the outcome of these patients. This prediction tool will be helpful to clinicians for assessing the risk classification of IMN patients and to decide who need more aggressive treatments and more frequent follow-up.

## Methods

### Study population and study design

All the patients in this study were recruited at Shanghai Ruijin Hospital from 2009.01 to 2013.12. The inclusion criteria were as follows: (1) renal biopsy was required for the diagnosis of IMN; (2) age ≥ 15 years; (3) informed consent was obtained. The exclusion criteria were: (1) patients with secondary causes of membranous nephropathy, such as malignancy, autoimmune disease and hepatitis B. (2) Patients receiving immunosuppressive treatment before hospitalization in our nephrology service. (3) Patients with severe heart failure or hepatic failure.

The primary outcome was defined as a combination of renal function progression, ESRD or death. Renal function progression was defined as a reduction in eGFR greater than or equal to 30% compared with renal function at the time of renal biopsy [15]. ESRD was defined as the need for dialysis or kidney transplant.

### Data collection

The patients’ demographic characteristics, baseline and follow-up clinical data were collected. eGFR was calculated by eGFR-EPI formula [16]. Renal biopsies were evaluated and scored by 2 experienced nephropathologists. The IMN stages was assessed based on the criteria listed below. Pathological staging oF IMN [17].

Stage I: Normal glomerule under light microscope (LM) with subepithelial immune complex deposits under electronic microscope (EM). Stage II: Heterogenous thickening of glomerular basement membrane (GBM) with formation of spikes under LM and subepithelial immune complex deposits under EM. Stage III: Evident thickening of GBM under LM. Either deposition of immune complex in the subepithelial space or in GBM is observed under EM. Stage IV: Evident thickening of GBM under LM. Thick GBM with absorption of immune complex is observed under EM. Severe interstitial fibrosis was defined as superior to 50% interstitial fibrosis. Serum PLA2R antibodies were measured by an ELISA test (Euroimmun, Lübeck, Germany) in patients whose serum was available at the time of renal biopsy. Glomerular PLA2R deposits were measured by using an indirect immunofluorescence test with anti-PLA2R antibody (Atlas Antibodies AB, Stockholm, Sweden). IgG subclasses were tested by direct immunofluorescence methods with mouse anti-human IgG1 FITC, anti-human IgG2 FITC, anti-human IgG3 FITC and anti-human IgG4 FITC (Southern Biotech, CAT. No 9200-02; Southern Biotech, CAT. No9080-02; Southern Biotech, CAT. No 9052-02; Southern Biotech, CAT. No 9210-02). PLA2R staining and IgG subclasses were evaluated by pathologists with standard immunofluorescence microscopy. The presence of granular capillary loop staining in the glomeruli was defined as positive.

### Statistical analysis

Continuous variables that were normally distributed were expressed as the mean ± SD and compared with Student’s *t*-test. Continuous variables that had a skewed distribution were presented as the median (Range) and compared with the Mann–Whitney U test. Categorical variables were compared with the Chi squared test. The proportional hazard assumption was checked by testing covariate-by-time interactions for each variable. The Cox proportional hazards models were built to test the associations between the variables and the outcomes. The best model was selected using a stepwise selection of variables using Akaike information selection criterion. Variables with P values less than 0.05 in the univariate analysis were included in the multivariate Cox proportional hazards models. A nonparametric bootstrapping resampling analysis with replacements was used to validate the risk factors obtained in multivariate Cox analysis. Only the independent predictors validated by bootstrapping resampling analysis were retained in the final model for the calculation of risk score. ROC curve were generated to compare the discrimination among the risk factors and risk scores. Two sided P value < 0.05 was considered statistically significant. The statistical analysis was performed with IBM SPSS (version 21.0, Chicago, IL, USA) and R software.

## Results

### Baseline demographic and clinical data

**Fig. 1:**
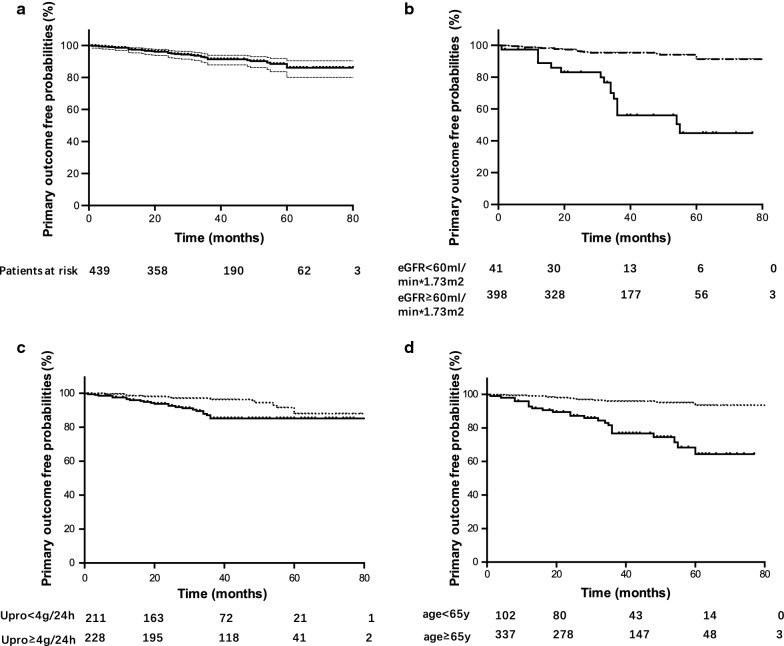
Survival curves for primary outcomes **a** in all IMN patients (n = 439): primary outcome-free time: 38.73 ± 19.35 months; **b** Solid lines: eGFR-EPI < 60 ml/min*1.73 m^2^, dashed lines: eGFR-EPI ≥ 60 ml/min*1.73 m^2^. In patients with eGFR-EPI ≥ 60 ml/min*1.73 m^2^ vs eGFR-EPI < 60 ml/min*1.73 m^2^: primary outcome-free time: 39.13 ± 19.22 months vs 34.85 ± 20.36 months, P = 0.18; HR: 0.12(0.06–0.23), P < 0.01; **c** Solid lines: urine protein ≥ 4 g/24 h, dashed lines: urine protein < 4 g/24 h. In patients with urine protein < 4 g/24 h vs urine protein ≥ 4 g/24 h: primary outcome-free time: 42.14 ± 19.37 months vs 35.04 ± 18.67 months, P < 0.01; HR: 3.89(1.18–12.79), P = 0.03; HR: 2.35(1.19–4.65), P = 0.01. **d** Solid lines: ≥ 65 years, dashed lines: < 65 years. In patients aged < 65 years vs ≥ 65 years: primary outcome-free time: months 39.24 ± 19.17 vs 37.04 ± 19.90 months, P = 0.32; HR: 6.15(3.12–12.14), P < 0.01; *Upro* urine protein

In total, 439 patients were recruited. The baseline characteristics of the enrolled patients are listed in Table 1. The median age of all the enrolled patients was 56 (15–83) years old with a male predominance sex ratio: male vs female was 1:0.91. Among all patients, baseline serum albumin was 23(8–43) g/l, eGFR was 100.31(12.81–155.98) ml/min/1.73 m^2^ and proteinuria was 3.98(1.50–22.98) g/24 h. Serum PLA2R antibody was measured in 130 patients with a median titer of 26.54 (0.52–1040.18) RU/L. A total of 59.91% of the patients were diagnosed as stages II IMN, 19.36% I IMN, 20.05% III IMN and 0.68% IV IMN (Additional file 1: Table S1). Compared to patients in other stages, patients in stage IV had heavier proteinuria, lower albumin and worse renal function at diagnosis. Severe tubulointerstitial lesions(account for ≥ 50%) were detected in 3.42% patients at the time of diagnosis. Renal PLA2R staining was positive in 85.11% patients. Of all patients with IgG subclasses testing, 84.82% were IgG1 positive, 58.04% IgG2 positive, 23.21% IgG3 positive, and 92.86% were IgG4 positive. During a median follow-up of 38.73 ± 19.35 months, 36 patients (8.20%) had primary events of which there were 24 (5.47%) renal function progression, 3 (0.68%) ESRD and 9 (2.05%) death (Table 1 and Fig. 1a).

**Table 1:** Baseline characteristics of IMN patients

	All
Follow-up time (m)	38.73 ± 19.35
Age (years)	56 (15–83)
Female (%)	209 (47.61%)
Albumin (g/l)	23 (8–43)
Proteinuria (g/24 h)	3.98 (1.5–22.98)
eGFR (ml/min/1.73 m^2^)	100.31 (12.81–155.98)
Microscopic hematuria (%)	260 (59.22%)
Triglyceride (mmol/l)	2.35 (0.70–10.92)
Cholesterol (mmol/l)	7.17 (1.84–16.71)
Uric acid (μmol/l)	357.03 ± 83.38
Serum PLA2R antibody (RU/l)	26.54 (0.52–1040.18)
Pathology	
LM-stages I and II (%)	348 (79.27%)
LM- ≥ 50% tubulointerstitial lesions (%)	15 (3.42%)
IF-PLA2R positive staining (%)	80/94 (85.11%)
IF-IgG1 positive (%)	95/112 (84.82%)
IF-IgG4 positive (%)	104/112 (92.86%)
Primary outcomes (%)	36 (8.20%)
Renal function progression (%)^a^	24 (5.47%)
Death (%)	9 (2.05%)
ESRD (%)	3 (0.68%)

Continuous variables presented as mean ± SD or median (range)

*ESRD* end-stage renal disease, *LM* light microscopic, *IF* immunofluorescence

^a^Renal progression: a reduction in eGFR greater than or equal to 30% compared with that at renal biopsy

### Identification of risk factors and establishing risk scores

The proportional hazard assumption was checked by testing covariate-by-time interactions for each variable (Additional file 1: Table S2) showing all these variables agreed with proportional hazard assumption. In the univariate analysis, age, serum albumin, proteinuria, eGFR and severe interstitial fibrosis were associated with primary outcomes. In multivariate analysis, the best model included age (HR: 1.04, 95% CI 1.003–1.08, P = 0.04, 95% CI from bootstrapping: 1.01–1.08), eGFR-EPI (HR: 0.97, 95% CI 0.96–0.99, P < 0.01, 95% CI from bootstrapping: 0.96–0.99) and proteinuria (HR: 1.09, 95% CI 1.01–1.18, P = 0.03, 95% CI from bootstrapping: 1.02 ~ 1.16). A risk score based on the regression coefficients of these 3 risk factors was then developed: Risk score = 0.04*Age (years) − 0.03*eGFR-EPI (ml/min/1.73 m^2^) + 0.09* proteinuria (g/24 h). Each unit increasing in the risk score was associated with a 2.57 (1.97–3.36) fold increasing in the risk of primary outcome occurrence (Table 2).

**Table 2:** Cox proportional hazards ratio model of primary outcomes

	Univariate analysis HR (95% CI)	P	Multivariate analysis HR (95% CI)	P	HR (95% CI) from Bootstrap analysis	P
Age (years)	1.09 (1.05–1.12)	< 0.01	1.04 (1.003–1.08)	0.04	1.04 (1.01–1.08)	0.01
Female	0.62 (0.31–1.21)	0.16	–	–	–	–
Albumin (g/l)	0.90 (0.85–0.95)	< 0.01	–	–	–	–
Proteinuria (g/24 h)	1.14 (1.06–1.21)	< 0.01	1.09 (1.01–1.18)	0.03	1.09 (1.02–1.16)	< 0.01
eGFR (ml/min/1.73 m^2^)	0.96 (0.95–0.97)	< 0.01	0.97 (0.96–0.99)	< 0.01	0.97 (0.96–0.99)	< 0.01
Microscopic hematuria	0.78 (0.40–1.50)	0.45	–	–	–	–
Triglyceride (mmol/l)	1.16 (0.97–1.39)	0.11				
Cholesterol (mmol/l)	1.06 (0.93 ~ 1.22)	0.39	–	–	–	–
Uric acid (μmol/l)	1.00 (0.99–1.01)	0.20	–	–	–	–
Serum PLA2R antibody (RU/l)	1.00 (0.99–1.00)	0.28	–	–	–	–
Pathological characteristics			–	–	–	–
Stages I and II	1.00 (0.46–2.22)	0.99	–	–	–	–
≥ 50% interstitial fibrosis	6.12 (2.54–14.73)	< 0.01	–	–	–	–
PLA2R staining positive	26.08 (0.00–3 × 10^6^)	0.59	–	–	–	–
IgG1 positive	0.99 (0.11–9.50)	0.99	–	–		
IgG4 positive	0.40 (0.04–3.63)	0.40	–	–		

### Assessment of the risk score performance

**Fig. 2:**
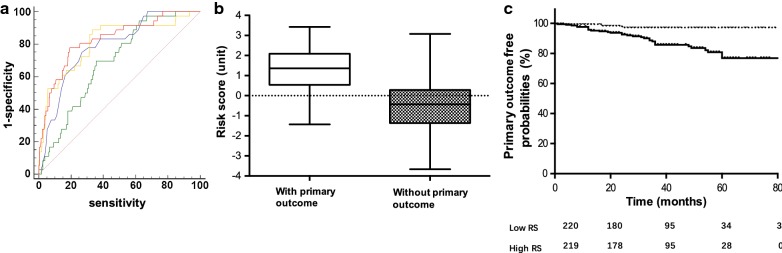
Comparison of ROC curves, comparison of risk scores and survival curve for primary outcomes (**a**). Red line: Risk score; yellow line: eGFR-EPI; blue line: age; green line: proteinuria. Risk score AUC: 0.83 (95% CI 0.76–0.90); age AUC: 0.79 (95% CI 0.72–0.86), eGFR-EPI AUC: 0.81(95% CI 0.73–0.89) and proteinuria AUC: 0.69(95% CI 0.61–0.77) (**b**).Comparison of risk scores between patients with and without primary outcome (− 0.43 vs 1.37, P < 0.01). **c** Survival curve and Kaplan–Meier analysis for primary outcomes by risk score: high risk score vs low risk score: HR: 5.97, 95% CI 2.32–15.35 (cut-off value: − 0.29). *RS* risk score

The ROC curve was generated to compare the prognostic values of the identified risk factors with the risk scores. The risk score (C statistics: 0.83, 95% CI 0.76–0.90) was better than each of the 3 risk factors alone including age(C statistics: 0.79, 95% CI 0.72–0.86), eGFR-EPI (C statistics: 0.81, 95% CI 0.73–0.89) and proteinuria (C statistics: 0.69, 95% CI 0.61–0.77), which suggested that the risk score was had better discrimination in predicting adverse outcomes in IMN patients (Fig. 2a). Besides, we compared the predicted risk versus observed rate of primary outcome. The discriminative slope was calculated as the difference between the mean predicted probability between IMN patients with or without primary outcome (1.80) indicating good performance of the risk score (Fig. 2b). Next we divided the patients into 2 groups based on median of risk scores at − 0.29. A Kaplan–Meier curve revealed that the patients in the high risk subgroup had a 5.97-fold increased risk for developing primary outcomes (HR: 5.97, 95% CI 2.32–15.35) compared with patients in the low risk subgroup (Fig. 2c).

### Associations between serum PLA2R antibody, renal PLA2R staining and primary outcome

There was no significant difference in serum PLA2R antibody titer (30.40 RU/L vs 12.17 RU/L, P = 0.09) and renal PLA2R positive staining (100% vs 84.27%, P = 1.00) between patients with or without primary outcome occurrences. Then, the patients were divided evenly into two groups (high vs low serum PLA2R antibody groups) based on median serum PLA2R antibody titers. A Kaplan–Meier curve revealed a similar tendency toward primary outcomes in the two groups (P = 0.19). No significant difference was observed in the occurrence of primary outcomes in patients with positive or negative renal PLA2R staining.

## Discussion

The disease course of IMN is quite variable. An effective tool for clinicians to decide which patients need more aggressive therapy and more frequent follow-up would be helpful in clinical practice. However, the risk factors reported by previous studies [11, 12] are still controversial, and there is no well-established consensus. Besides, most of the studies were conducted in western countries and needed to be validated in other populations, such as Asian populations. Thus, our study has explored the risk factors of adverse outcome in 439 IMN patients by Cox proportional hazards model and validated by bootstrap resampling analysis. Then we developed a risk score based on the 3 independent risk factors (age, eGFR and proteinuria) retained in the final Cox multivariate model. The risk score showed a good discriminating based on ROC curve. One unit increasing of the risk score was associated with 2.57 (1.97–3.36) fold increasing risk of primary outcome. To our knowledge, this is the first risk prediction tool based on baseline parameters that has been proposed for risk stratification in IMN patients and is helpful to improve clinical practices.

In our study, we found that age, eGFR and proteinuria were 3 independent risk factors for unfavorable outcome in IMN patients. The associations between age and ESRD in IMN patients was controversial based on previous studies. Shiiki et al. [3] enrolled 949 Japanese IMN patients and found that male gender, older age (≥ 60 years), higher serum creatinine concentration (≥ 1.5 mg/dl) and tubulointerstitial changes were associated with ESRD. However, no correlation was found between age and renal progression in Zent’s study [14]. He found although the mortality rate was higher, the ESRD rate was similar in elderly IMN patients than in young patients. It’s probably due to the competing risk of death and renal progression that masked the effect of age on renal outcome. Our study confirmed the correlation between age and a combined outcome consisting of renal function progression, ESRD and death. The associations between creatinine, proteinuria and inferior renal outcome in IMN patients were reported by several studies [8, 18]. In our study, we validated these findings in Asian population by using eGFR-EPI instead of creatinine which is a better way in evaluating baseline renal function.

In our study, we did not validate the correlations between baseline serum anti-PLA2R antibody levels, renal PLA2R antigen and adverse outcomes in IMN patients. Kanigerchela et al. [19] found that high level of PLA2R antibody was correlated to disease activity and a higher risk for renal function deterioration based a study of 90 IMN patients. More recently, studies [9, 10] suggested that dynamic measurement of serum anti-PLA2R antibodies was helpful to predict treatment response and relapses. Considering the limited number of patients with serum anti-PLA2R antibody and renal PLA2R antigen measurements in our study, a more extended study is needed to draw further conclusions.

Cattran et al. has proposed a predictive model of renal prognosis in IMN patients based on dynamic changes of proteinuria and creatinine [20–22]. This model need follow up patients for a period of time before the risk stratification. In our study, we constructed a risk predicting tool based on baseline parameters, which can be directly applied into newly diagnosed IMN patients. The risk score showed a good discrimination based on ROC curve and discriminative slope between IMN patients with or without primary outcome. Thus, our risk score will be useful for clinicians to perform risk stratification for IMN patients.

Our study has several limitations. An external cohort of more ethnically diversified patients is needed for further validation. In addition, our study was based on a retrospective review of IMN patients, which needed to be further evaluated in a prospective cohort.

## Conclusion

Our study indicated that older IMN patients with lower eGFR and heavier proteinuria at the time of renal biopsy were at a higher risk for having adverse outcomes. A new risk score based on these three variables provides clinicians an effective tool for risk stratification.

## Additional file

**Additional file 1: Table S1.** Baseline characteristics of different grades of IMN patients. **Table S2.** Hazards proportional assumption test by COX time-dependant covariates.

## Abbreviations

IMN: idiopathic membranous nephropathy
ESRD: end-stage renal disease
eGFR: estimated glomerular filtration rate
PLA2R: anti-phophalipase A2 receptor
