# Supplementary material for: New risk score for predicting progression of membranous nephropathy
Source: J Transl Med. 2019 Feb 8;17:41. doi: 10.1186/s12967-019-1792-8 (PMC6368730; doi:10.1186/s12967-019-1792-8)
Supplement: Supplementary file 1 — Additional file 1: Table S1. Baseline characteristics of different grades of IMN patients. Table S2. Hazards proportional assumption test by COX time-dependant covariates. [file 12967_2019_1792_MOESM1_ESM.docx]

Table S1. Baseline characteristics of different grades of IMN patients

|  | Stage I | Stage II | Stage III | Stage IV | |
| --- | --- | --- | --- | --- | --- |
| No. | 85(19.36%) | 263(59.91%) | 88(20.05%) | 3(0.68%) |  |
| Age (years) | 51(15~74) | 57(15~83)^a^ | 57(17~79)^a^ | 57(26~74) |  |
| Follow-up (months) | 37.89±18.32 | 38.02±18.02 | 41.70±23.78 | 36.67±14.57 |  |
| Female (%) | 49(57.65%) | 121(46.00%) | 39(44.32%) | 0 |  |
| Albumin (g/l) | 27(12~41) | 22(8~43)^a^ | 20(12~35)^ab^ | 20(19~29) |  |
| Proteinuria (g/24 h) | 3.07(1.50~15.35) | 4.28(1.59~20.89)^a^ | 4.34(1.60~22.98)^ab^ | 8.93(3.95~9.50)^abc^ |  |
| eGFR(ml/min/1.73m2) | 108.90(55.84~154.87) | 99.83(12.81~152.51)^a^ | 90.53(14.31~155.98)^a^ | 47.18(42.33~76.85)^ab^ |  |
| Microscopic hematuria (%) | 39(45.88%) | 168(63.88%)^a^ | 50(56.82%) | 3(100%) |  |
| Triglyceride (mmol/l) | 2.22(0.88~9.17) | 2.26(0.70~10.92) | 2.58(0.70~9.06) | 5.13(1.44~6.21) |  |
| Cholesterol (mmol/l) | 6.23(1.84~14.50) | 7.45(2.56~16.71)^a^ | 7.58(3.52~15.35)^a^ | 5.61(5.42~5.68) |  |
| Uric acid (umol/l) | 341.35±71.68 | 359.22±86.86 | 365.55±81.17 | 360.00±131.82 |  |
| Serum PLA2R antibody (RU/L) | 13.53(0.92~296.57) | 30.18(1.03~1040.18)^a^ | 35.83(0.52~562.94) | 31.85(24.21~39.50) |  |
| Pathology |  |  |  |  |  |
| *LM- tubulointerstitial lesions≥ 50% (%)* | 0(0%) | 7(2.66%) | 8(9.09%)^ab^ | 0 |  |
| *IF-PLA2R positive staining（%）* | 17/21(80.95%) | 51/59(86.44%) | 12/14(85.71%) | 0 |  |
| *IF-IgG1 positive（%）* | 20/26(76.92%) | 63/72(87.50%) | 12/14(85.71%) | 0 |  |
| *IF-IgG4 positive（%）* | 24/26(92.31%) | 67/72(93.06%) | 13/14(92.86%) | 0 |  |
| Outcomes (%) |  |  |  |  |  |
| *Renal function progression* (%)* | 3(3.53%) | 18(6.84%) | 3(3.41%) | 0 |  |
| *Death (%)* | 1(1.18%) | 5(1.90%) | 3(3.41%) | 0 |  |
| *ESRD (%)* | 0(0%) | 1(0.38%) | 2(2.27%) | 0 |  |

Note: continuous variables presented as mean± SD or median (range); Abbreviations: ESRD: end-stage renal disease；LM: light microscope; IF: immunofluorescence; * Renal progression: a reduction in eGFR greater than or equal to 30% compared with that at renal biopsie

a: compared with stage I, P<0.05

b: compared with stage II, P<0.05

c: compared with stage III, P<0.05

Table S2. Hazards proportional assumption test by COX time-dependant covariates

|  | HR(95% CI) | P |
| --- | --- | --- |
| Age (years) | 1.06(0.95~1.18) | 0.33 |
| Female | 0.47(0.04~5.36) | 0.54 |
| Albumin (g/l) | 0.89(0.73~1.09) | 0.24 |
| Proteinuria (g/24 h) | 1.03(0.79~1.34) | 0.85 |
| eGFR(ml/min/1.73m2) | 0.97(0.93~1.01) | 0.09 |
| Microscopic hematuria | 0.06(0.003~1.13) | 0.06 |
| Triglyceride (mmol/l) | 0.98(0.50~1.92) | 0.96 |
| Cholesterol (mmol/l) | 0.95(0.57~1.57) | 0.83 |
| Serum PLA2R antibody (RU/L) | 0.99(0.96~1.03) | 0.80 |
| Pathological characteristics |  |  |
| Stages I&II | 1.87(0.14~24.25) | 0.63 |
| ≥ 50% interstitial fibrosis | 7.45(0.40~139.14) | 0.18 |
| PLA2R staining positive | 25.37(0.0001~2*10^20) | 0.89 |
| IgG1 positive | 0.54(0.001~431.40) | 0.86 |
| IgG4 positive | 7*10^15(0.0001~3*10^40) | 0.75 |
